# Zebrafish aggression on the sub-second time scale: evidence for mutual motor coordination and multi-functional attack manoeuvres

**DOI:** 10.1098/rsos.180679

**Published:** 2018-08-15

**Authors:** Andres Laan, Marta Iglesias-Julios, Gonzalo G. de Polavieja

**Affiliations:** Champalimaud Neuroscience Programme, Champalimaud Center for the Unknown, Lisbon, Portugal

**Keywords:** aggression, evolutionary game theory, decision rules

## Abstract

Most animals fight by repeating complex stereotypic behaviours, yet the internal structure of these behaviours has rarely been dissected in detail. We characterized the internal structure of fighting behaviours by developing a machine learning pipeline that measures and classifies the behaviour of individual unmarked animals on a sub-second time scale. This allowed us to quantify several previously hidden features of zebrafish fighting strategies. We found strong correlations between the velocity of the attacker and the defender, indicating a dynamic matching of approach and avoidance efforts. While velocity matching was ubiquitous, the spatial dynamics of attacks showed phase-specific differences. Contest-phase attacks were characterized by a paradoxical sideways attraction of the retreating animal towards the attacker, suggesting that the defender combines avoidance manoeuvres with display-like manoeuvres. Post-resolution attacks lacked display-like features and the defender was avoidance focused. From the perspective of the winner, game-theory modelling further suggested that highly energetically costly post-resolution attacks occurred because the winner was trying to increase its relative dominance over the loser. Overall, the rich structure of zebrafish motor coordination during fighting indicates a greater complexity and layering of strategies than has previously been recognized.

## 1. Introduction

Animals fight by roaring [1], lunging [2], circling [3], head-waving [4], head-butting [5], biting [6], wrestling [4] and by a myriad of other ways [7]. We have good theories and measurements about why an animal should start a fight with a fin display and end it with mouth wrestling [8], but we have less information about what exactly happens during a lunge, a circling display or a directed attack manoeuvre.

Studies in several other sub-fields of animal behaviour show that quantification of within-behaviour limb and body dynamics is critical to test scientific hypotheses. For example, high-speed cameras enabled measurements on fly leg movements during escape jumps. These data, in turn, helped in the discovery of a context-sensitive control system in what was previously believed to be a simple ballistic reflex [9]. Likewise, statistical descriptions of escape trajectories have provided evidence of protean behaviour—a strategy where prey occasionally randomize their movement direction in order to reduce the degree to which their behaviour can be predicted [10,11]. The analysis of peregrine falcon attack trajectories has revealed a mathematical analogy between falcon prey capture and ballistic missile targeting [12].

The last two examples are particularly relevant for the study of aggression, because they illustrate cases where a complete understanding of strategic behaviours requires ways to record the dynamics occurring within elementary behaviours. Next, we highlight some outstanding issues in the study aggression which might similarly benefit from modern data-capture methods.

During contests, zebrafish frequently engage in repeated attacks in which one animal performs a rapid directed movement towards another and the other animal sometimes responds with an avoidance/retreat manoeuvre [6,13,14]. What has remained unclear is the quantitative relationship between the attack manoeuvres and the avoidance manoeuvres. Does every attack induce an avoidance manoeuvre? Are the locomotor costs of an attack greater or smaller than the locomotor costs of a retreat?

These questions are relevant to correctly interpret zebrafish fights. Animal conflict is partly structured as a series of assessments of relative strength. Different game-theory models postulate different relationships between individual activity levels and fitness costs of assessment. War of attrition (WOA) models postulate that only signallers suffer costs [15–17]. Sequential assessment (SA) [18] and cumulative assessment (CA) [19] models also allow the signaller to have a direct effect on the receiver's fitness. To better analyse assessment strategies of zebrafish, we must find ways to quantify the costs of attacking and defending.

Measuring the fine structure of fights may also help to identify new domains in which evolutionary game theory can be tested. We have evidence that short-time-scale strategic behaviour shapes other competitive interactions like escape manoeuvres [10,11,20]. During fights, similar sub-games might unfold within the multitude of elementary interactions. In the last section of our results, we identify one such sub-game and develop a game-theory analysis of the process.

Analysis of elementary aggressive interactions can also shed light on multiple functions of a single behaviour. When a boxer holds up his hands, it is with the dual purpose of being ready to both attack and defend. Likewise, a zebrafish attack may be shaped by multiple competing requirements of defence, display and offence. Without large-scale datasets, it is difficult to experimentally assess which of the many behaviours might serve multiple functions [14].

To address these open questions, we developed methods to analyse zebrafish fights at a high resolution. We took inspiration from several pre-existing machine learning tools to create a system which allows for tracking and identifying unmarked animals as well as automatically annotating their behaviours [21–23]. The resulting system provides the user with trajectory data containing information about velocities, accelerations and relative positions of the fighting individuals as well as an automated ethogram which identifies the behaviour performed by any animals at any given moment.

## 2. Material and methods

### 2.1. Staging of contests

We used 68 male zebrafish of the AB strain and of approximately 1 year of age. All holding rooms were ventilated through a centralized HVAC system and were kept at controlled room temperature (25°C), 50–60% humidity. Fish holding rooms were kept under a 14 L : 10 D cycle with a light intensity of 200–300 lx at the water surface. The density of the fish in the tanks was 10 fish l^−1^, and in a typical cage we had 20–25 animals. Our general feeding protocol consisted of two types of live feeds, rotifers and *Artemia* nauplii, and a processed dry feed (Gemma Micro, Skretting, Spain). Depending on the fish age, the feeding frequency varied. In the months prior to experiments, the fish were fed Gemma500 feed and live de-capsulated *Artemia* once a day.

We staged contests between zebrafish by adapting a procedure from Oliveira *et al.* [6]. A pair of males were removed from their home tanks and kept in visual but not olfactory isolation for a period of between 24 and 48 h. In a slight departure from Oliveira *et al.* [6], the fight was staged in an arena which was different from and larger than the arena used for pre-fight isolation to avoid the confounding influence of walls on swimming behaviour, which occurs too frequently in small arenas. The fight was staged in a uniform rectangular arena with dimensions 32 × 24 × 12 cm, slightly rounded corners and water depth of approximately 7 cm. Care was taken to ensure a lack of sharp illumination gradients in the tank so as to facilitate later tracking. Recordings began when the two animals were simultaneously poured from the isolation tank into the fight arena. A typical recording lasted for 1 h and was continued for another hour in the rare cases when the fight appeared unresolved after 1 h. After the fight was terminated, both animals were returned to their home tanks. Video data were acquired at 20 frames per second using Matlab standard functions.

For manual annotation of behaviour, we followed [6,13]. Attacks were detected as events in which one individual made a rapid and directed swim towards another individual, sometimes accompanied by mouth opening and biting, to which an opponent may respond either by a retreat manoeuvre or by a sudden acceleration followed by a flip of the tail.

Dominance in our set-ups is dynamic and can switch suddenly at certain points. Our operational definition was that a fish is counted as dominant during a time period if it delivers more than 90% of the attacks during the previous 4 min.

### 2.2. Tracking

Aggressive contests in zebrafish pose three challenges. First, fighting is a three-dimensional process and the manoeuvres are facilitated by deep waters, which induces appearance changes as the depth of the fish varies. Second, fish change their appearance not only due to varying depth but also due to colour changes during the fight. Third, collisions are more frequent during fighting than during schooling. This motivated the use of a hybrid system in which a new version of idTracker [21] (idtracker.ai [24]) using deep convolutional networks was used for tracking when the animals were not colliding, because of the greater expressive capacity of learned templates compared with the hand-engineered template of classical idTracker. When the pair of fish collided, a Gaussian mixture model was used to separate the colliding animals (as in [23]) and identity information was propagated into the collisions by using a greedy acceleration minimization principle along the trajectory with the constraint that identities of both trajectories at the start and end of the collision had to be matched with the predictions from idtracker.ai (see [21] for an analogous algorithm for collision resolution).

The greedy acceleration minimization was implemented step by step. At each time step, two candidate coordinates (each representing the centre of mass of a fish with unknown identity) originating from the GMM algorithm needed to be identified. We considered the identities of the coordinates at the previous two time steps to be fixed and then we calculated the absolute net linear acceleration along both trajectories for the two possible identity assignments. Whichever assignment resulted in the lower total acceleration was used for final identification and the cycle was repeated again and again until the end of collision. During collisions, we had an identification accuracy of 98%.

### 2.3. Automated behaviour classification and analysis

To improve data efficiency, we used a preprocessing method which was designed to reduce translational and rotational variance. We dynamically transformed our data series into a new coordinate system in which the zero was located at the joint centre of mass of the pair of fish at time *t* − *K*. The *x*-axis was aligned with a vector which pointed from fish 1 towards fish 2 at time *t* − *K*. All coordinates of the four vectors were converted into this coordinate system. After the preprocessing, the four processed vectors were then concatenated into a single vector and passed as input to the first layer of a standard multi-layer perceptron with a ReLu hidden layer activation function, a cross-entropy loss function. Using this preprocessing and an amount of annotated data that was small compared with the total corpus of data analysed, we were able to train a perceptron with two hidden layers of size 250 neurons to have a test set accuracy of 95%. In the electronic supplementary material, §1.2.2, we describe further control analysis to prove that our method selectively targets aggressive displays and not just general social behaviour (see also electronic supplementary material, figure S1).

All further analysis was done using custom-written Matlab code. The forcemap technique was used from Katz *et al.* [25]. At each point in time, we used a focal fish-centric coordinate representation where the focal fish was looking along the *y*-axis. The focal fish acceleration vector was decomposed into components that were perpendicular (turning force) and parallel (speeding force) to its velocity. Then a map of the average force as a function of partner fish location was computed. In order to avoid potential influences from walls, the symmetric phase forcemaps were analysed only when both fish were further than 5 cm away from the nearest wall. During the asymmetric phase, the fish spent most of their time swimming very close to the wall, but we excluded the influence of corners on turning by removing all data when fish were closer than 5 cm to the nearest corner.

## 3. Results

We staged 34 contests between pairs of adult male zebrafish (see Material and methods for details). To analyse the fine details of the contests, we developed a custom tracking system. We used idtracker.ai [24] to track and identify unmarked animals, and we added to it a custom-written collision resolution system to resolve the identities of the two animals when they collide (see Material and methods for machine learning procedures). The output of the tracking system is a time series of trajectories for both contestants at a sampling rate of 20 Hz.

**Figure 1.**
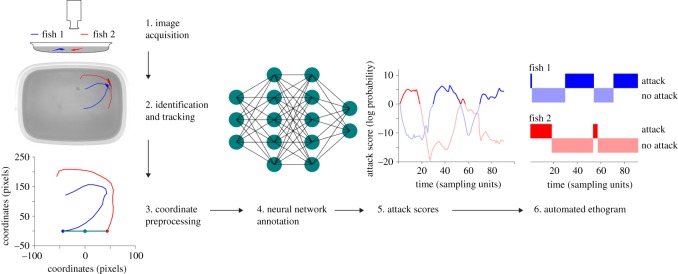
Computer vision pipeline. 1: Raw video. 2: Unmarked animals after identification with idtracker.ai and a short span of the trajectory of each animal overlaid. 3: Preprocessing of a local portion of trajectory for neural network analysis. 4: Schematic of the neural network classifier which was trained to mimic human annotations. 5: Time series of attack scores for two animals as produced by the neural network classifier. High attack score values indicate a high internal confidence of the network that an attack is taking place. 6: Automatic ethogram calculated by thresholding the attack score.

We then manually annotated a small fraction of our video data to indicate when attacks were taking place. These annotations were then used to train a neural network to detect the presence of attacks from trajectory data with 95% test set accuracy. By combining several augmented and improved machine learning tools into a common pipeline, we created software that automatically provides information about the movement, behaviour and identity of each animal on a sub-second time scale (figure 1).

### 3.1. Analysis of activity correlations and assessment models

**Figure 2.**
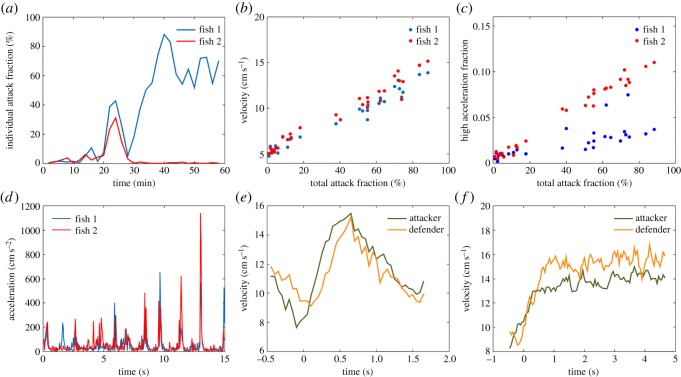
Kinematic characterization of a fight. (*a*) Fraction of an animal's time budget used in attacks (individual attack fraction) over the course of the fight (blue and red curves mark the two different individuals here and elsewhere). Analysis is conducted in non-overlapping 2 min time windows. Minutes 24–28 correspond to the symmetric phase and minutes 34–60 to the asymmetric phase. (*b*) Correlation between total attack fraction (sum of individual attack fractions) and velocity. (*c*) High-intensity acceleration bouts (*a* > 128 cm s^−2^) occur mostly during attacks. (*d*) Time series of acceleration during the symmetric phase of the fight depict the occurrence of sudden acceleration bouts. (*e*) Velocity of attacker (green) and defender (orange) during an average attack in the symmetric phase (*N* = 114). Attacks begin at time 0 and the termination of the plot coincides with the median attack duration in the symmetric phase. See electronic supplementary material, figure S3, for average acceleration plots. (*f*) Same as (*e*) but for the asymmetric phase (*N* = 116).

We first characterized the large-scale patterns of aggressive behaviour in our dataset. We found clear signs of aggression in 27 of the 34 staged contests. Fights consisted of two distinct phases (figure 2*a*; see also electronic supplementary material, video S1). During what we called the symmetric phase (figure 2*a*, 24–28 min), both individuals engaged in mutual attack behaviour. During the asymmetric phase, mainly one individual performed attack behaviours (figure 2*a*, 34–60 min). When a symmetric phase was present (*N* = 15 fights), the most common pattern (*N* = 8 of 15 fights) consisted of a pre-fight phase with very few attacks (figure 2*a*, 0–22 min), followed by a symmetric phase, in turn followed by an asymmetric phase in which only one individual engaged in attacks. It is thus likely that the symmetric phase is similar to the contest phase described in many other model systems of aggression, whereas our asymmetric phase resembles the resolution phase [26,27].

In the next sections, our analysis will focus on 14 of the 15 fights in which the symmetric phase was present unless stated otherwise (with one fight excluded from the analysis since its long duration posed a threat to animal welfare and had to be stopped; see also electronic supplementary material, §1.2.3 and figure S2, for further description of fight types).

We used the outputs of our machine learning pipeline to analyse velocity and acceleration as two coarse kinematic parameters of attacks. We focused on these variables first since they can be regarded as an approximate individual measure of energy expenditure [28]. Attacks in both the symmetric and asymmetric phases had higher velocities than the pre-fight phase. Pre-fight, the fish had an average speed of 5.3 ± 0.84 cm s^−1^ (*N* = 13, mean ± standard deviation), which during the symmetric phase rose to 10.3 ± 1.9 cm s^−1^ (*N* = 14) for the attacker and 10.9 ± 1.4 cm s^−1^ (*N* = 14) for the defender. We note that, here and elsewhere, the roles of attacker and defender were not fixed during the analysis of a fight but were calculated dynamically for each individual at each moment in time based on the outputs of our classifier. Fish swimming speed rose further during the asymmetric phase with 13.5 ± 1.6 cm s^−1^ (*N* = 11) for the attacker and 14.0 ± 1.6 cm s^−1^ (*N* = 11) for the defender. As a further check of our analysis, we binned data from each fight into consecutive non-overlapping 2 min long segments and calculated the average speed and the total percentage of time that attacks were occurring (the total attack fraction) during each time bin. There is a strong linear correlation between average movement speed and attack percentage (figure 2*b*; *r* = 0.90 ± 0.06, *N* = 28 individuals).

Fighting is associated not only with an increase in velocity but also with bursts of high acceleration (figure 2*d*). As with speed, there was a strong correlation between the total attack fraction and the total fraction of time each animal spent in acceleration bursts (figure 2*c*; *r* = 0.88 ± 0.15, *N* = 28).

These results are consistent with attacks inducing a strong energetic cost for both attacker and defender. This point is reinforced if we time-align individual attacks and calculate the average velocity waveform for both the attacker and the defender during both the symmetric (figure 2*e*) and the asymmetric (figure 2*f*) phase. From figure 2*e,f*, it is apparent how attacks begin with an increase in the velocity of both the attacker and the defender. In fact, the locomotor costs for the defender is on average higher as they swim with a higher average speed in 20 out of the 25 conflict phases analysed (*p* = 0.004, two-tailed binomial test). These findings are compatible with the assumptions of the CA and SA models and violate the assumptions of WOA models (see electronic supplementary material, table 1.1.1, for a summary comparison of the different models).

We compared our approach with established methods of analysis that recommend disambiguating assessment strategies by studying the covariation between resource holding potential (RHP; fighting ability) and the duration of the contest phase (the symmetric phase in our terminology). The first step involved finding an indicator of RHP. In our dataset, size was a statistically significant indicator of RHP as the larger animal ended up as the dominant individual in 20 out of 25 fights where we could identify a clear winner (*p* = 0.002, one-tailed binomial test, analysis includes fights both with and without a symmetric phase). In fights where a symmetric phase was present, there was a statistically significant trend for large size differences to be associated with shorter fights (*r* =−0.47, *p* = 0.045, one-tailed *t*-test for Pearson's correlation coefficient). A linear regression analysis of the effects of the sizes of both contestants yielded a model where the larger individual's size had a negative effect on fight duration and the smaller individual size had a weaker but positive effect on fight duration, although the latter value was not significantly different from zero (*c*_large_ =−0.14, *c*_small_ = 0.04, *p*_large_ = 0.02, *p*_small_ = 0.52).

A negative relationship between body mass difference and fight time is expected in all three models (WOA, CA and SA) [29]. A negative effect of larger individual body size on fight duration is incompatible with a WOA model of contest behaviour. Our result that the size of the larger individual has a stronger effect on fight times than the body size of the smaller individual is inconsistent with a pure SA game model. However, it is in principle consistent with the CA model (see electronic supplementary material, Mathematical analysis of the cumulative assessment model).

We also found that the symmetric phase was associated with systematic changes in fish body colour, but these changes were not reliably correlated with the identity of the winner (see electronic supplementary material, Methods and figure S4).

### 3.2. Analysis of movement rules

Previously, studies [6,13] had described zebrafish attacks as locomotion manoeuvres in which the attacker orients its body towards the defender and then swims rapidly towards it. The defender typically responds by swimming away from the attacker in a manoeuvre named retreat. Additionally, the attacker sometimes veers to the sides of the defender in order to deliver bites to the sides of the defender. Based on this description, we had four baseline expectations. First, the attacker is expected to be located behind the defender most of the time. Second, the attacker is expected to exhibit an acceleration response towards the defender if the defender is in front of the attacker. Third, the defender is expected to exhibit a repulsive speeding response when the attacker is behind it (the running away response). Fourth, when the attacker is located to one side (e.g. the right) of the defender, the defender was expected to turn towards the other side (e.g the left) in order to dodge potential bites. However, the following analysis of the movement rules shows that the last three expectations are only partly correct.

**Figure 3.**
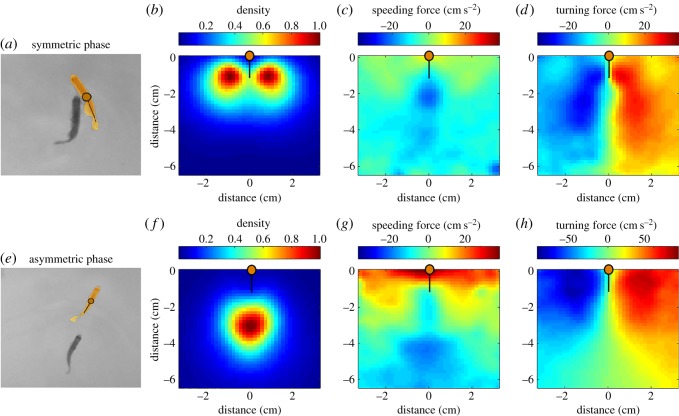
Forcemaps of the defender in the symmetric (*a–d*) and asymmetric (*e–h*) phase. (*a*) Typical configuration of the two fish during the symmetric phase. (*b*) Probability density map of the position of the attacker relative to the defender during the symmetric phase. Note that the negative numbers in the distance axis indicate that the attacker is behind the defender. (*c*) Speeding force of the defender as a function of the relative location of the attacker during the symmetric phase. Red colour corresponds to speeding up and blue to slowing down. (*d*) Turning acceleration of the defender as a function of the relative location of the attacker during the symmetric phase. Red colours correspond to turning to the right and blue to the left. (*e*) Typical configuration of the two fish during the asymmetric phase. (*f*–*h*) Same as (*b*–*d*) for the asymmetric phase. All maps averaged over *N* = 14 fights, 230 000 time points.

**Figure 4.**
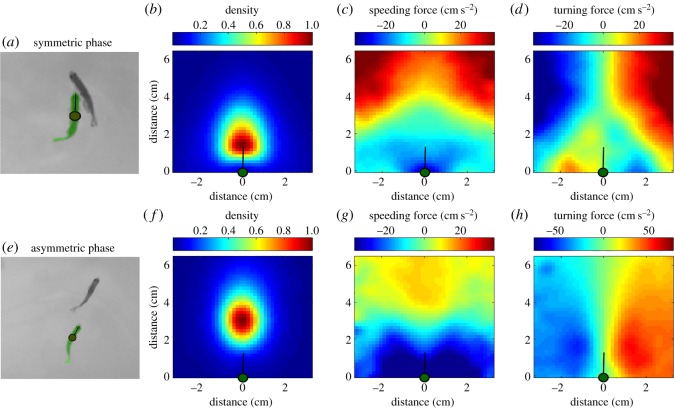
Forcemaps of the attacker in the symmetric (*a–d*) and asymmetric (*e–h*) phase. (*a*) Typical configuration of the two fish during the symmetric phase. (*b*) Probability density map of the position of the defender relative to the attacker during the symmetric phase. (*c*) Speeding force of attacker as a function of the relative location of the defender during the symmetric phase. Red colour corresponds to speeding up and blue to slowing down. (*d*) Turning acceleration of the attacker as a function of the relative location of the defender during the symmetric phase. Red colours correspond to turning to the right and blue to the left. (*e*) Typical configuration of the two fish during the asymmetric phase. (*f*–*h*) Same as (*b*–*d*) for the asymmetric phase. All maps averaged over *N* = 14 fights, 230 000 time points.

We characterized the kinematic movements from the point of view of a focal animal [25,30,31]. We used a coordinate system in which the focal fish is located at the centre of the coordinate system and is looking up along the *y*-axis (depicted as an orange dot in figure 3 for defender and as a green dot in figure 4 for attacker; see Material and methods for details).

We begin by considering the defender as the focal fish and analyse the positions of the attacker with respect to the defender (figure 3*b,f*). These figures give the distribution of the locations of the attacker relative to the defender during attacks in the symmetric (figure 3*b*) and asymmetric phases (figure 3*f*). In both, the attacker is typically located behind the defender. During the symmetric phase, the attacker is located about half a body length behind the defender and is positioned to the left or to the right of the defender (figure 3*b*). By contrast, during asymmetric phase attacks the attacker is typically located a whole body length behind the defender (figure 3*f*).

We then analysed the acceleration of the focal fish in terms of the location of the partner, known as forcemap analysis [25,30,31]. We started with the attacker as the focal fish and computed its speeding response (speeding is defined as acceleration parallel to the focal fish's own velocity vector) depending on the position of the defender (figure 4*c,g*). When the defender is far in front of the attacker, the attacker tends to accelerate towards the defender (red and yellow areas at the top of the maps in figure 4*c,g*). However, the expectation of pure attraction was violated at close range by the presence of repulsion zones (blue areas at the bottom of figure 4*c,g*). If the attacker reached close to the defender, there was a tendency for the attacker to decelerate rather than accelerate. This deceleration response was present even if we removed periods of collision from the analysis (see electronic supplementary material, figure S5C, bottom panel), making unlikely an explanation based on some direct physical interaction such as contact-driven repulsion.

The speeding map of the attacker was consistent with a strategy in which the attacker maintains a constant distance from the defender by speeding towards the defender if the defender was far away and by slowing down and letting the defender escape if the defender was too close. This finding was at odds with our initial expectation that the primary goal of the attacks was to create bodily contact which would enable delivery of bites. Additionally, we also found evidence for such a distance-maintaining strategy by the defender. The speeding map of the defender in both phases showed acceleration responses (running away) when the attacker was too close (figure 3*c,g*) and deceleration responses (permitting approach) when the attacker was far away.

We then examined the turning acceleration of the attacker as a function of the location of the defender (figure 4*d*). When the defender was far away, the attacker exhibited a turning response towards the defender. However, at close range, the turning response once again changed to a repulsive response (notice the flipped polarity at the bottom of the turning map when compared with the top in figure 4*d*).

The turning map of the defender during the symmetric phase showed, in contrast to our initial expectation, that it turns towards the side of the attacker instead of away from it (figure 3*d*). If the attacker was, say, to the left of the defender, the defender turned to the left towards the attacker. This response by the defender contributed to the stable maintenance of the T-like configuration the two fish often make during the symmetric phase (figure 3*a*). Our initial hypothesis was that the T-configuration resulted from efforts by the attacker to swim to this position so that he could deliver biting attacks to the vulnerable sides of the opponent. Examination of the forcemaps supports more the idea that the defender contributes to the maintenance of the T-configuration by exhibiting a tendency to turn towards the attacker, thus exposing the sides of its body even further. Contact is instead avoided because the attacker decelerates in response.

The T-configuration is persistent, as shown by the strongly peaked nature of the position histogram (figure 3*b*). This observation speaks against our initial hypothesis of the T-configuration as a non-persistent state arising through counter-manoeuvres designed to avoid the bites. The balance of evidence indicates that the T-configuration during symmetric phase attacks may instead be a ritualistic configuration which is maintained by mutual efforts and may thus function partly as a mutual display-like behaviour.

Consistent with the display hypothesis, the T-configuration did not appear during post-resolution attacks—presumably because display behaviours are superfluous after the winner has been resolved. As can be seen from figure 3*e,f*, the typical configuration during post-resolution attacks had the attacker located behind the defender and the defender running away from the attacker in a straight line. This pattern is maintained due to the distance-maintaining strategy in the speeding maps. Furthermore, the turning response of the defender in the region where the attacker is most likely to be located has a green zone of neutrality (figure 3*h*, green triangle-like area at the bottom of the map) rather than an attractive turning response as was seen in the symmetric phase. This finding supports the idea that, in the post-resolution phase, the defender simply avoids the attacker rather than engaging in a more complex strategy.

While the maps presented in figures 3 and 4 used pooled data from the 14 conflicts which exhibited a symmetric phase, individual fights show very similar maps (electronic supplementary material, figures S6 and S7). These maps are thus a reliable feature of zebrafish aggression.

**Figure 5.**
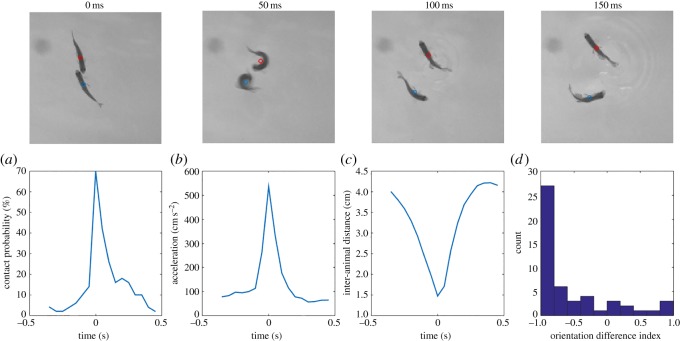
The splash behaviour. Top panels: a time-lapse series of four consecutive frames (sampled at 50 ms) during an example splash behaviour. Bottom panels: (*a*) probability of contact during a splash. (*b*) Average acceleration of an individual during a splash. The splash takes place at time 0. (*c*) Average time evolution of inter-animal distance during a splash. (*d*) Histogram of the orientation change index during 50 acts of splash. An index of −1 corresponds to a 180° degree change in orientation; an index of 1 corresponds to orientation unchanged (see Material and methods for details). For all plots, *N* = 50 splash behaviours. Owing to their short duration and rare occurrence, the splash behaviours were found by manual annotation.

We also found evidence for a novel type of aggressive manoeuvre which we termed ‘splash’ (figure 5, top panels). We named the behaviour splash after the characteristic ripple pattern which forms on the water surface after the manoeuvre is performed. The splash behaviour typically took place as one zebrafish approached another and, as the two made contact (figure 5*a*), one or both of them responded with a sudden acceleration manoeuvre (figure 5*b*) which resulted in the orientations of the two fish being completely reversed and the two fish being propelled apart by a distance of a few body lengths or more (figure 5*c*). Typically, a 180° change in orientation (figure 5*d*) was completed in less than 50 ms. We believe the splash may be key to stabilizing the display-like attacks (see Discussion).

### 3.3. Modelling of the asymmetric phase

We were motivated to seek a theoretical treatment of the post-conflict phase by the observation that both the winner and the loser engage in contact-free but high-velocity chases, which are costly for both the winner and the loser. Why does the winner use a strategy which results in wasted energy on attacks even after it has established its dominance in the symmetric phase? Based on previous studies [26,32,33], we hypothesized that the winner implements a strategy which simultaneously damages the loser without risking the possible loss of dominance.

We can, therefore, think of the post-resolution fight as a zero-sum game, in which the reward for the winner is *r*_*w*_ = *C*_*l*_ − *C*_*w*_ and the reward for the loser is *r*_*l*_ = *C*_*w*_ − *C*_*l*_ (*C*_*w*_ and *C*_*l*_ designate the costs incurred by the loser and the winner, respectively). What are the possible ways that two fish can impose costs on one another? Zebrafish incur costs either through rapid swimming or by receiving bites from the opponent. During the post-resolution phase, the dominant engages in rapid swimming with an approximately constant velocity (figure 2*f*) while staying a constant distance away from the subordinate (figure 3*f*). Our analysis must explain why the dominant never accelerates enough to touch and deliver bites to the opponent, which would certainly help to selectively reduce subordinate fitness.

In order to maintain a stable velocity *v*, the winner fish must generate a force *F*(*v*), which carries a cost *C*(*F*). If the loser also swims with velocity *v*, it will also incur a cost *C*(*F*). If the winner engages in biting, it will deliver to the loser an additional cost *C*_*b*_. However, it is reasonable to expect that the opportunity to deliver bites at velocity *v* will also not come without a cost to the attacker. It must produce extra force in order to generate some pressure between its own mouth and the body of the loser. In addition, extra energy may be needed for moving the jaws and potentially suffering a less streamlined posture because of the bending needed to deliver the bites. The extra force needed, *δF*, will induce a greater cost of *C*(*F* + *δF*), while the loser incurs a cost of only *C*(*F*).

For real fish, the functions *F*(*v*) and *C*(*F*) are obviously not completely generic. *F*(*v*) is monotonically increasing because higher velocities require higher forces in order to overcome increased drag. The function *C*(*F*) is likely to be not only monotonically increasing but also convex, since maintaining higher forces requires recruitment of more energetically inefficient muscle groups [34]. For convex functions, Δ*C* = *C*(*F* + *δF*) − *C*(*F*) is also an increasing function of *F*. At equilibrium, it must be the case that the winner cannot increase its reward by switching from steady chasing at velocity *v*_*e*_ and force *F*(*v*_*e*_) to a biting attack at velocity *v*_*e*_ and force *F* + *δF*. Therefore, at equilibrium, Δ*C*(*F*) = *C*_*b*_. Since Δ*C*(*F*) is an increasing function of *F*, there exists a value of *F* high enough for this condition to be true. Another solution to the model involves limits on the range of possible values of *F*. If *F* has a biological maximum, *F*_max_, which is smaller than the value of *F* when biting costs become equal to attack costs, then the equilibrium value *v*_eq_ is given by *F*(*v*_eq_) = *F*_max_ − *δF*. In both cases, the equilibrium has a stable value of *v*_eq_.

From our theoretical analysis, we also conclude that it is necessary for the loser fish to maintain a high velocity *v*_eq_ because otherwise biting attacks become profitable for the winner and the loser will have further reduced relative capacity. The winner, in turn, must maintain a high velocity and a close distance to its opponent or else the loser may respond by slowing down since the dominant fish is too far away to attack. The caudal deceleration zones apparent in the speeding map of the defender in the asymmetric phase (figure 3*g*) are compatible with such strategic responses. The analysis thus indicates a plausible link between game-theory equilibria, well-known features of fish muscular physiology, and the observed long duration chasing which often concludes zebrafish fights.

## 4. Discussion

We have introduced a machine vision pipeline for the study of aggression in zebrafish which allows both automated identification and tracking of unmarked animals by use of idtracker.ai [24] as well as individual-level automated classification of ethologically relevant behaviours on a sub-second time scale (other systems do not identify individuals and/or use engineered features instead of deep learning [22,23]). The pipeline allows for reduced human workload by elimination of marking and annotation stages as well as reducing the need for controls comparing marked and unmarked animals. Our methods also have the additional advantage of allowing for some parallelization. Though we focused here on experiments in large arenas to avoid the confounding influence of walls on our analysis, it is possible to fit up to four smaller fight arenas into the field of view of our camera. The tracking can also be done in parallel without modifications to the code. Hence, it is feasible for certain experiments to increase the set-up throughput by a factor of 4.

One potential deficiency in our current method is the inability to reliably monitor fin movements in large arenas due to camera resolution limitations. Fins are commonly used in aggressive displays and an ability to automatically monitor their activity could provide further information about contest dynamics. With the improving quality and resolution of cameras, it may soon be possible to build set-ups in which fish are simultaneously recorded from all three directions and computer vision is used to reconstruct fin activity.

In our work, we have also demonstrated how the ability to gather high-resolution trajectory data can help to decide which of the many assessment models gives the best description of the fight. For example, we observed a strong correlation between the velocity of the attacker and the defender during individual attacks. The observation of strong mutual correlation in activity levels during individual acts of behaviour gives evidence that, in zebrafish, approximately equal locomotor costs are borne by both the producer of the attack as well as its target. This observation rules out WOA models of contests as good descriptions of zebrafish aggression. We reach this conclusion because these models posit individual behavioural acts to have an effect on the energy budget of only the one that is producing the signal and not on the target of the signal [15]—a hypothesis clearly violated in our data.

Based on our results, the standard SA game also appears ill-suited as a description of zebrafish aggression because we were unable to detect a statistically significant positive relationship between the fight time and the RHP of the loser [29]. Having ruled out both the self-assessment and the SA models, we were left by elimination with the CA game as the only suitable description of zebrafish fighting and our supplementary modelling also supported this conclusion.

We believe our approach is a valuable complement to the current standard methodology of game-theory model testing in two contexts. First, as others have argued [4] and as we showed in our supplementary modelling, the distinction between the CA and WOA models in terms of fight time scaling relationships is not as clear-cut as is sometimes stated [29,35]. In such cases, the use of machine learning tools to infer activity budgets and correlations from video data may become a valuable complement to the standard toolkit as it will occasionally allow resolution of the ambiguities. Second, since our analysis does not require knowledge of the RHP, it can be used in cases where the RHP is unknown or RHP differences are small.

Beyond the falsification of game-theory models, analysis of trajectory-level data also proved useful in clarifying the nature of certain behaviours. In the beginning, we believed that the primary function of attacks in the symmetric phase was to manoeuvre the attacker into a position where it might be able to elicit further damage through direct contact and biting. We were surprised to find in our forcemaps that, rather than avoiding such attacks, the defender had a statistical tendency to turn its flank towards such attacks. Even more surprisingly, the attacker had a tendency to incompletely exploit the resulting vulnerable configuration, as evidenced by the presence of weak repulsion zones in the turning rule of the attacker. The willingness of the defender to expose its flank may thus be at least partly a display behaviour intended to signal its ability to manoeuvre and/or withstand damage.

The potential risk of the display may be mitigated by the opportunity to engage in the splash behaviour. The splash behaviour may enable one fish to halt or perturb the approach of another as it is becoming too dangerous. In support of this, notice how the splash is usually deployed right as the attacker is making first contact with the defender. The potential option to engage in the splash behaviour may also mitigate the risk associated with engaging in the display-like attacks, which leads the defender into a vulnerable configuration. The vulnerable configuration which occurs during the displays may be stabilized because the attacker knows that any attempt to exploit the vulnerability can be countered with a splash manoeuvre by the defender.

We introduced the use of movement rules [25] to the analysis of contests. The ability to quantify the fine structure of aggressive attacks through movement rules is useful not only for the insight it provides about typical fighting tactics, but also because it enables quantification of change in those tactics. There is now much evidence for the role of cognition and learning in shaping animal fighting ability [36]. Fighting ability of animals changes with experience [37,38], but exactly how experience makes fighters more competent and skilful has not always been clear from the studies. It may be that changes in fine motor dynamics play an important role and our measurement toolbox could be helpful in clarifying some of these unresolved issues. For example, evidence from sticklebacks has established a role for learning in the development of displays [39]. If the same is true for zebrafish, then there is an expectation that, early in development, contest phase attacks might lack some of the display-like features we see an adults. The forcemap technique we have introduced could be straightforwardly applied to address this hypothesis, which might prove more difficult to test with traditional methods.

In the last section, we introduced a new game-theory model of post-assessment fighting behaviours. The resulting model helped explain why fish engage in costly chases (which appear to equally drain the resources of both participants) even after the dominance status has been resolved. Furthermore, the model proved a good explanation for the form of movement rules adopted during that phase. The model makes a series of further predictions which can be tested in future experiments. For example, the model predicts that, as bites become more damaging, the speed of chases is expected to increase. This prediction could be tested in the fgfr1a mutant, which exhibits poorly formed scales and is, therefore, expected to be more vulnerable to biting attacks [40,41].

Finally, we hope that the study of trajectory-level data will help to open new avenues in the study of strategic conflict. With the ability to record high-resolution data, we may be able to get a better understanding of the biomechanical determinants [31] of movement during contests. This may finally allow us to study the long-ago stated goal of examining not just how displays are used, but what factors determine the form and also the fine dynamics of the displays [42]. Or in other words, we may eventually be able to study the movement sub-games taking place within the larger assessment games. We took a small step in that direction by explaining the qualitative patterns of locomotion during the chasing phase through a game-theory analysis, but there is also a need for better theoretical methods to analyse the extended games which occur when acceleration decisions influence inter-individual distances over time. The recent merging of techniques from game theory and deep reinforcement learning represents a promising avenue for further research in this regard. In particular, the use of self-play, which has allowed humanoid robots to teach each other wrestling in an unsupervised way, is a technology which could be applicable to the study of fish aggression [43].

## Supplementary Material

Supplementary models and figures

## Supplementary Material

Video S1

## Data accessibility

A video containing example instances of fighting behaviour has been uploaded as part of the electronic supplementary material. The code and data associated with this publication have been uploaded to Dryad under http://dx.doi.org/10.5061/dryad.142q2vb [44].
