# Supplementary material for: Zebrafish aggression on the sub-second time scale: evidence for mutual motor coordination and multi-functional attack manoeuvres
Source: R Soc Open Sci. 2018 Aug 15;5(8):180679. doi: 10.1098/rsos.180679 (PMC6124137; doi:10.1098/rsos.180679)
Supplement: Supplementary models and figures [file rsos180679supp1.pdf]

Supplementary for: Zebrafish aggression on the sub-second time-scale- evidence for mutual motor coordination and multi-functional attack maneuvers

July 10, 2018

1 Supplementary materials

1.1 Mathematical analysis of the cumulative assessment model

In the cumulative assessment model, animals accumulate costs as a result of their own activity as well as the activity of their opponent. Therefore the rate of cost accumulation is a function $f(q_1, q_2)$ of both q_1 and q_2 which denote the resource holding potentials of the first and the second animal (assume $q_1 < q_2$). The total damage animal 1 is willing to accept before surrender is a function of only his own RHP and we express it as $g(q_1)$. The fight time is given as $t = \frac{g(q_1)}{f(q_1,q_2)}$. If the variation in qualities is small we can write both f and g as Taylor series, which, if we keep only the linear terms, will yield the equation $t = K\frac{1+\alpha_1 q1}{1+\alpha_2 q_1+\alpha_3 q_2}$, where the normalization constant K is used to normalize the baseline values of f and g.

The correlations observed between t, q_1 and q_2 depend on the value of the coefficients $\alpha_1, \alpha_2, \alpha_3$. For α_1, the only biologically plausible value range would be for $\alpha_1 > 0$, since higher quality should translate to a greater ability to persist despite accumulating damage. However, this does not necessarily mean that increases in the value of q_1 increase the fight times. It may be the case that the value of α_2 exceeds the value of α_1. Then, the ability to accept greater costs with increasing quality is overwhelmed by simultaneous tendency of higher quality individuals to expend more energy when attacking

(such a trade-off may be rational if attacks are particularly damaging to the opponent). Thus, depending on the values of α_1, α_2, q_1 may have a positive or negative effect on fight times.

The case of α_3 is easier to analyze. A higher quality opponent should result in more damage elicited with each attack or a greater attack rate. α_3 should therefore be positive and q_2 should be negatively correlated with fights times, which is the case we observe in our data. As one final point, we note that if $\alpha_3 = 0$, then we are left with only the quality/RHP of the looser as the determinant of the fight duration. The specific case is important, because a dependence of fight times on only the RHP of the looser is widely taken to be an indicator of the WOA model. Our analysis thus points to further ambiguities that occur when one tries to determine assessment models solely based on analysis of RHP and fight time covariation. It also further motivates the utility of our new technique in circumventing these ambiguities by testing the underlying assumptions of the different assessment models directly.

1.1.1 Tabular summary of assessment models

Assessment model	Cost structure	Fight time scaling	Escalation
WOA	Signaler pays cost of producing signal	Loser body mass increases fight time, winner body mass irrelevant	Escalation is allowed within a phase
SA	Signaler and receiver can both bare direct costs	Loser body mass increases, winner body mass decreases fight times	Escalation only between phases
CA	Signaler and receiver can both bare direct costs	Diverse outcomes possible	Escalation is allowed within a phase

1.2 Supplementary methods

1.2.1 Measurement and analysis of color changes

The easiest way to estimate color changes is to calculate the average intensity of each fish identified fish whose silhouette has been separated from the background by thresholding the image intensity. However, this approach brings with it certain biases, because the arena is not uniformly illuminated. The area near the walls in particular tends to have a stronger shadow than the

central arena. Since fighting fish distribute themselves near the walls during the asymmetric phase and near the center during the symmetric phase, use of the raw intensity risks confounding the effects of location and intrinsic intensity change.

In order to remove the bias, we used linear regression to dissociate the effects of time and space on fish intensity. The rectangular arena was divided into a 6-by-6 grid and each grid rectangle was associated with a regression coefficient. Time likewise was partitioned into 2 minute long segments and each segment associated with a regression coefficient. For each fight and each fish, we carried out a separate linear regression between the fish intensity, the location and time. We used the regression coefficients associated with time as indicators of the reflectance change of each fish.

A linear regression model was used because of the following fact of physics. Reflected illumination is the product of incident light intensity $I(x, y)$ which in the setup depends on position and not on time, and the reflectance of the fish r(t), which evolves over time but not over space. Overall fish intensity C is given as $C = I(x, y)r(t)$. If we assume that the changes in reflectance and incident light intensity are small, then color change at any given time and place is well approximated as $\Delta C(x, y, t) = r_{mean}\Delta I(x, y) + I_{mean}\Delta r(t) + \Delta I(x, y)\Delta r(t) \approx r_{mean}\Delta I(x, y) + I_{mean}\Delta r(t)$, which is linear in both reflectance and illumination.

As mentioned in the main text, another weak predictor of fight outcome was color. We found that zebrafish exhibited a transient darkening which occurred specifically during the symmetrical contest phase (see **Figure S4**). On average, the symmetric fight phase was accompanied by an $8\% \pm 4\%$ ($N = 28$) darkening of appearance in both fighters and this transient largely disappeared irrespective of whether the fight ended with asymmetric chasing or not. The eventual looser tended to darken more than the winner. In 9 out of 10 fights, the eventual looser had a higher intensity change relative to pre-fight intensity than the eventual winner ($p = 0.02$, 2-tailed binomial test). However, color change was a weak predictor of how the fight ended, since unequal changes in color were also associated with fights that ended without a clear way to determine the winner because chasing behavior was absent.

1.2.2 Classifier validation by sociality analysis

One potential concern for the use of our classifier is that rather than detecting aggressive behavior specifically, it instead detects social behavior in general. This may happen because general social behavior such as schooling shares many of the same features that attack behavior does, including

close inter-individual distance and alignment of the interacting individuals. We therefore examined how well our classifier tracks the so-called sociality index (Miller and Gerlai, Beh. Brain Res. 2007, Hinz and Polavieja, PNAS 2017). The sociality index compares the average inter-individual distance (d_a) during some time period with a permuted distance (d_p), where the spatial coordinates of the two individuals have been shuffled with respect to time (i.e. the permuted trajectories represent hypothetical fish that still have the same place preferences but do not coordinate their movements with each other). The sociality index is calculated as $SI = \frac{d_p - d_a}{d_p}$. The sociality index is close to 1 when fish are interacting with each other in an attractive fashion, whereas it is nearly zero when fish do not show social interactions.

In **Figure S1** we plot how the sociality index as well as an index of individual aggression for four different fights evolves over time. During the pre-conflict period, our fish displayed a variety of different behaviors. In some fights, the pre-conflict phase was characterized by schooling behavior (see **Figure S1** first 10 minutes in the two top panels), which is evidenced by a high sociality index. Note also that the classifier did not confuse this social behavior with true aggression since attack fractions of both individuals remained low during the same period and only rose later near the 20 minute mark. In other sessions, the fish spent parts of the pre-conflict period freezing or swimming in a non-social fashion (see for example **Figure S1** bottom right panel first 10 minutes), which is evidenced by the low sociality scores occurring at those times. This indicates that the sociality scores in our setups showed great variability and the lack of a strong correlation between sociality scores and fight scores could not be attributed to the fact that sociality scores do not vary in our experiments. Therefore our classifier is able to successfully tell apart aggression from other types of social and non-social behaviors.

1.2.3 Description of fight types

Not all fights followed the progression from pre-fight to symmetric to asymmetric (resolution) phase. In some cases, the symmetric phase was not followed by an asymmetric phase and in others, an asymmetric phase both proceeded and followed the symmetric phase. Interestingly, the individual who was dominant before the symmetric phase was not necessarily the one who attacked after the symmetric phase (see **Figure S2** for examples plots of fights in the more rare cases). In 12 of 34 fights, the only phase present was the asymmetric one.

1.3 Supplementary figures

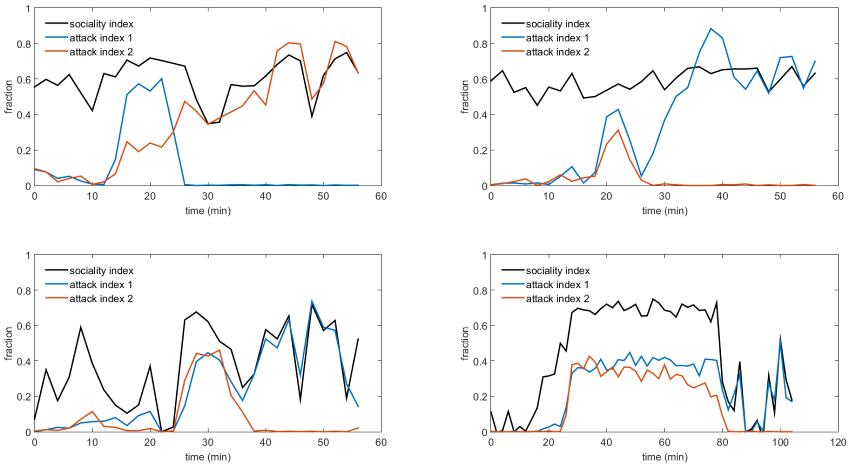

Figure S1: **The classifier selectively targets aggressive episodes**. We plot the sociality index (black trace) and the individual attack fractions of both animals (red and blue traces) for four different fights. As can be seen from the plots, the sociality index is a distinct measure which does not always correlate with the attack fraction (see for example the first 10 minutes in the top panels, where sociality is high but attack fraction stays low).

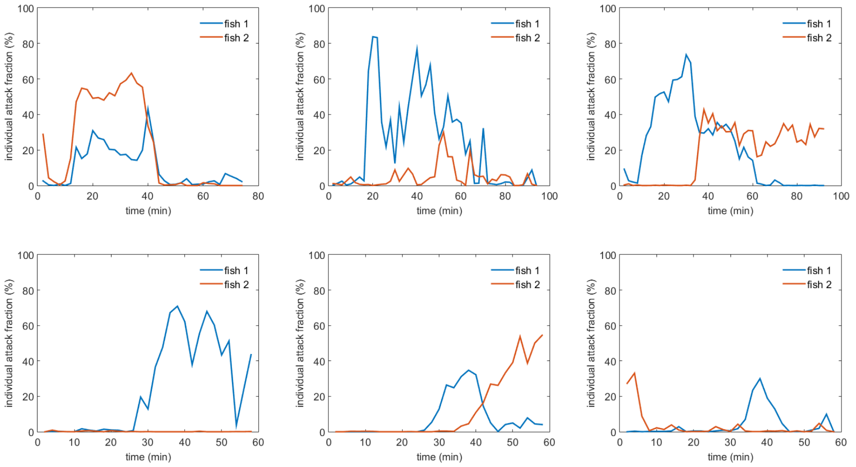

Figure S2: **An illustration of variability in fight dynamics**. A plot of the time series of attack rates for 6 different fights. Top left: a fight with a symmetric phase that ends without an asymmetric phase. Top middle: One animal predominantly attacks but the attack rate is irregular. Note the short duration symmetric phase around the 50 minute mark. Top right: A fight where the symmetric phase is both preceded and followed by an asymmetric phase.The animal who dominates in the beginning is not the eventual winner. Bottom left: a fight with only an asymmetric phase and one animal dominant. Bottom middle: a fight without a symmetric phase where the dominant individual switches in the middle of the fight without a symmetric phase. Bottom right: a fight with irregular and sporadic asymmetric attacks on both sides.

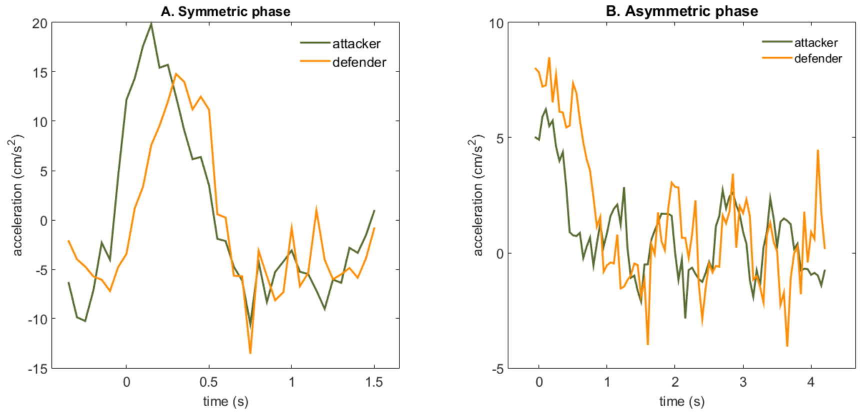

Figure S3: **Phase-typical acceleration waveforms**. A. The average acceleration of the attacker and the defender during an attack for the symmetric phase of the conflict (acceleration was obtained by numerically differentiating a velocity signal which was low-pass filtered by a moving average filter with kernel length 0.25 seconds). B. Same as A but for the asymmetric phase of the conflict

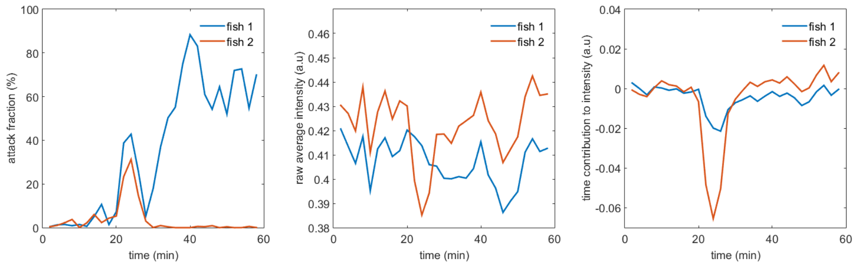

Figure S4: **Measuring changes in color**. Left panel: attack rates for the two animals over the course of the fight. Middle panel: raw average intensity of each animal over the course of the fight, the confounding influence of spatial variation in illumination has not been removed. Right panel: the change of intensity of both animals over time with confounding effects of spatial illumination inhomogeneity removed (see supplementary methods). The plots reveal a transient darkening which occurs in both animals during the fight. Notice the larger change in intensity in the eventual looser.

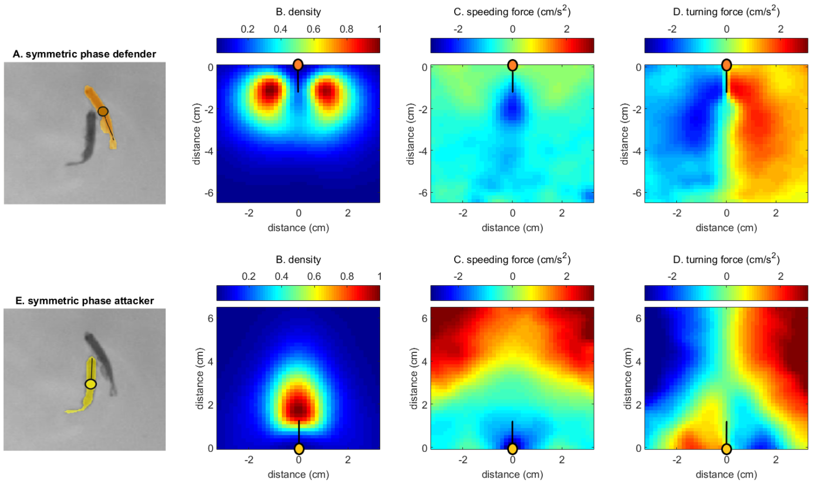

Figure S5: **Forcemaps of the symmetric phase with periods of collision removed**. Same maps as shown in main paper Figure 3 and 4 top row panels. They differ from how the maps in the main paper were calculated by the fact that we have removed the periods where the two animals were physically colliding. Top panels: Maps of the defender during the symmetric phase of the fight. Bottom panels: Maps of the attacker during the symmetric phase of the fight.

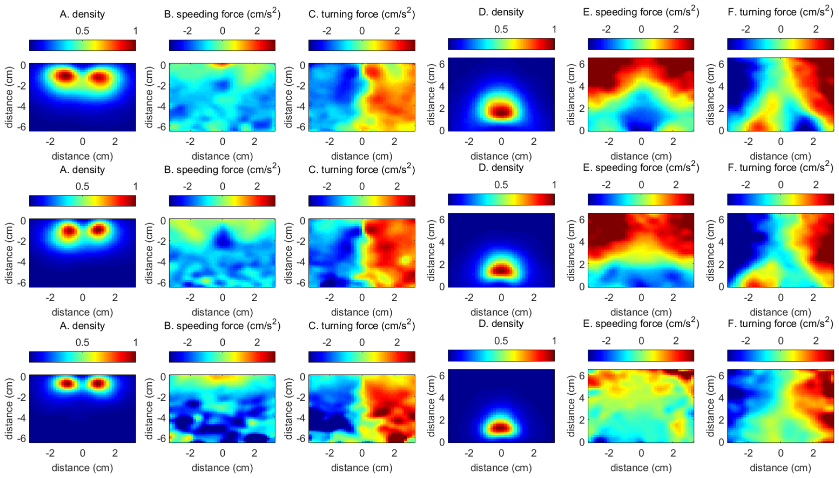

Figure S6: **Example symmetric phase forcemaps calculated based on single fights**. Each row depicts symmetric phase defender (A-C) and attacker (D-E) forcemaps for a different fight. A: defender location map B: defender speeding map C: defender turning map D: attacker location map E: attacker speeding map F: attacker turning map

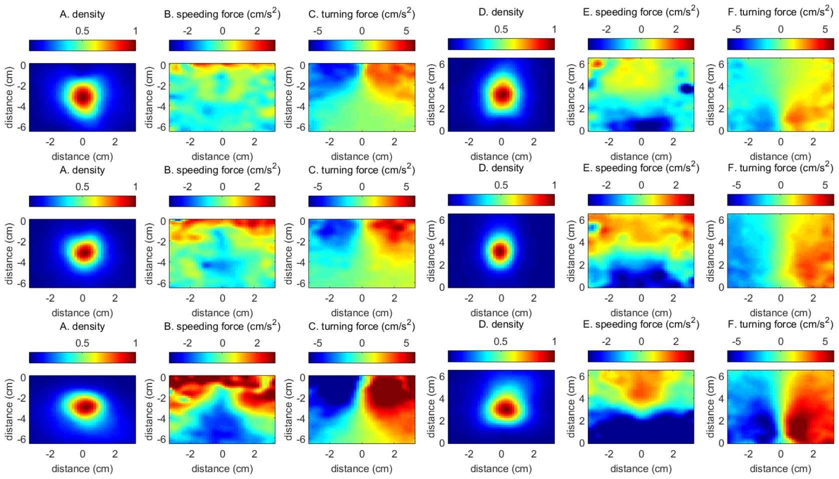

Figure S7: **Example asymmetric phase forcemaps calculated based on single fights**. Each row depicts asymmetric phase defender (A-C) and attacker (D-E) forcemaps for a different fight. A: defender location map B: defender speeding map C: defender turning map D: attacker location map E: attacker speeding map F: attacker turning map

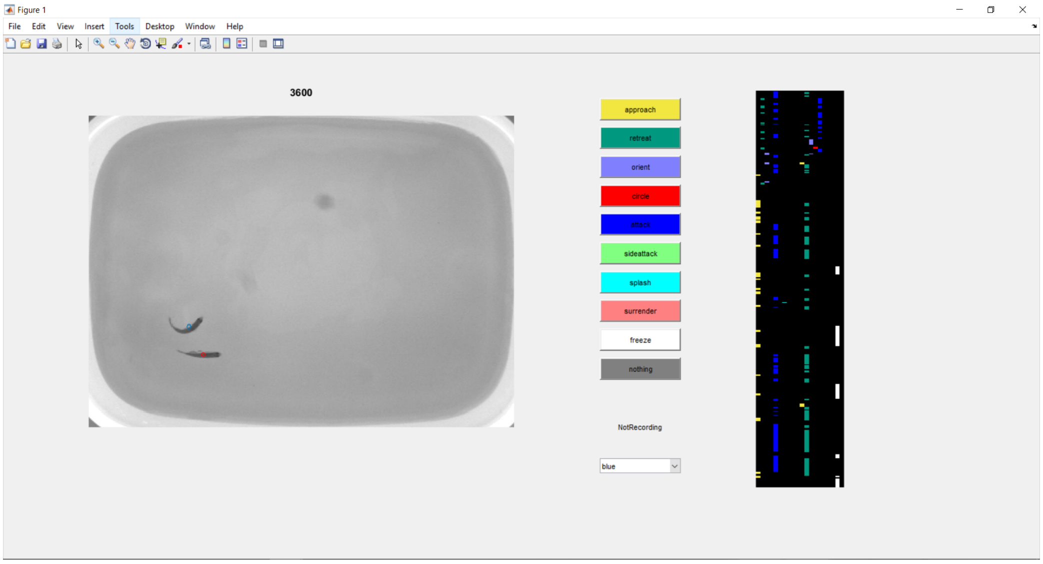

Figure S8: **An example frame of the GUI used to annotate videos**. The left panel shows a scrollable feed of the video which is used to examine the video frame by frame with controllable gain of scrolling. The middle panel displays a set of buttons to annotate behaviors and a menu to choose the focal animal. The right panel shows an ethogram which is dynamically updated as the investigator adds new annotations.
